# Lipoxin A_4_ Attenuates Constitutive and TGF-β1–Dependent Profibrotic Activity in Human Lung Myofibroblasts

**DOI:** 10.4049/jimmunol.1500936

**Published:** 2015-08-14

**Authors:** Katy M. Roach, Carol A. Feghali-Bostwick, Yassine Amrani, Peter Bradding

**Affiliations:** * Department of Infection, Immunity and Inflammation, Institute for Lung Health, University of Leicester, Leicester LE1 7RH, United Kingdom; and; † Division of Rheumatology and Immunology, Department of Medicine, University of South Carolina, Columbia, SC 29208

## Abstract

Idiopathic pulmonary fibrosis (IPF) is a common, progressive, and invariably lethal interstitial lung disease with no effective therapy. The key cell driving the development of fibrosis is the myofibroblast. Lipoxin A_4_ (LXA_4_) is an anti-inflammatory lipid, important in the resolution of inflammation, and it has potential antifibrotic activity. However, the effects of LXA_4_ on primary human lung myofibroblasts (HLMFs) have not previously been investigated. Therefore, the aim of this study was to examine the effects of LXA_4_ on TGF-β1–dependent responses in IPF- and nonfibrotic control (NFC)–derived HLMFs. HLMFs were isolated from IPF and NFC patients and grown in vitro. The effects of LXA_4_ on HLMF proliferation, collagen secretion, α-smooth muscle actin (αSMA) expression, and Smad2/3 activation were examined constitutively and following TGF-β1 stimulation. The LXA_4_ receptor (ALXR) was expressed in both NFC- and IPF-derived HLMFs. LXA_4_ (10^−10^ and 10^−8^ mol) reduced constitutive αSMA expression, actin stress fiber formation, contraction, and nuclear Smad2/3, indicating regression from a myofibroblast to fibroblast phenotype. LXA_4_ also significantly inhibited FBS-dependent proliferation and TGF-β1–dependent collagen secretion, αSMA expression, and Smad2/3 nuclear translocation in IPF-derived HLMFs. LXA_4_ did not inhibit Smad2/3 phosphorylation. In summary, LXA_4_ attenuated profibrotic HLMF activity and promoted HLMF regression to a quiescent fibroblast phenotype. LXA_4_ or its stable analogs delivered by aerosol may offer a novel approach to the treatment of IPF.

## Introduction

Idiopathic pulmonary fibrosis (IPF) is a progressive disease with a median survival of only 3 years (1, 2). Patients present with breathlessness and disabling cough, progressing to respiratory failure and a distressful death. The cause is not known, but alveolar cell injury coupled with fibroblast/myofibroblast proliferation and activation are critical in the pathophysiology (3, 4). There is little effective treatment, and there is therefore an urgent unmet clinical need for novel modulators of lung fibrosis and tissue remodeling.

The key cell driving the development of fibrosis is the myofibroblast (5, 6). Myofibroblasts are intermediate in phenotype between fibroblasts and smooth muscle cells, expressing α-smooth muscle actin (αSMA) and exhibiting contractile activity, but they are also the principle cell responsible for the synthesis and deposition of the fibrotic matrix in IPF (7). The increased numbers of myofibroblasts found within IPF lungs occurs in part through the differentiation of resident fibroblasts (8); this involves reorganization of the actin cytoskeleton, increased expression of αSMA, and incorporation of actin stress fibers (9), a process regulated by the TGF-β1/Smad pathway (10, 11). Furthermore, myofibroblasts derived from IPF lungs demonstrate enhanced proliferation (12), migration (13), collagen production (14), αSMA expression (15),and actin stress fiber formation (15). The myofibroblast is therefore a highly attractive target for the treatment of IPF. As myofibroblasts rarely persist in healthy lungs, their differentiation is considered a key event in the pathogenesis of IPF, and it is likely that a therapy capable of reversing this phenotypic change would be efficacious.

A family of lipid mediators known as lipoxins, resolvins, protectins, and maresins (collectively called resolving mediators for convenience) (16–18) are important for the resolution of inflammation and generated soon after a tissue insult. Lipoxins are generated from membrane arachidonic acid through biochemical synthesis involving the enzymes 5- and 15-lipoxygenase (5-LOX and 15-LOX) (18). Several molecules are generated through transcellular synthesis with 15-LOX active in one cell such as an epithelial cell and 5-LOX active in a second cell such as an inflammatory leukocyte. In addition, aspirin-triggered forms of these molecules exist (epi-lipoxins and aspirin-triggered resolvins) in which acetylated cyclooxygenase-2 generates the initial metabolite, which is then modified further by 5-LOX. Several molecules can also be formed endogenously in the absence of aspirin, possibly via a cytochrome P450-dependent pathway (17), and 5-LOX–independent pathways for the production of protectins and maresins also exist with unicellular synthesis evident (18).

A key feature of these molecules is that they promote resolution of inflammation at low nanomole concentrations, but are not innately immunosuppressive as they also activate antibacterial mechanisms (17, 19). Lipoxin A_4_ (LXA_4_) also has antifibrotic activity in a number of model systems. For example, it inhibits platelet-derived growth factor–dependent TGF-β1 production and profibrotic gene expression by renal mesangial cells (20), inhibits mesangial cell proliferation (21), and experimental renal fibrosis (22). LXA_4_ also inhibits epithelial mesenchymal transition in renal epithelial cells (23), whereas knockout of the 12/15-LOX pathway prevents experimental dermal fibrosis (24). With respect to lung fibrosis, LXA_4_ inhibited connective tissue growth factor-dependent proliferation of a human lung fibroblast cell line (25), whereas a stable epi-LXA_4_ analog reduced bleomycin-induced pulmonary fibrosis in mice (26).

The effects of LXA_4_ on the function of healthy and IPF-derived primary human lung myofibroblast (HLMF) function are unknown. We hypothesized that LXA_4_ inhibits constitutive HLMF profibrotic responses and TGF-β1–driven profibrotic activity through the Smad signaling pathway. We therefore investigated the effects of LXA_4_ on constitutive and TGF-β1–dependent HLMF Smad 2/3 activity, gene transcription, and profibrotic HLMF processes such as contraction, collagen secretion, proliferation, and differentiation.

## Materials and Methods

### Human lung myofibroblast isolation, characterization, and culture

Nonfibrotic control (NFC) HLMFs were derived from healthy areas of lung from patients undergoing lung resection for carcinoma at Glenfield Hospital, Leicester, U.K. No morphological evidence of disease was found in the tissue samples used for HLMF isolation. IPF HLMFs were derived from patients undergoing lung transplant at the University of Pittsburgh Medical Center and were shown to have usual interstitial pneumonia on histological examination. Myofibroblasts were grown, cultured, and characterized as previously described (27). All NFC patients gave informed written consent, and the study was approved by the National Research Ethics Service (references 07/MRE08/42 and 10/H0402/12). Written informed consent was also obtained from all IPF subjects, with the protocol approved by the University of Pittsburgh Institutional Review Board.

All cultures demonstrated the typical elongated spindle-shaped fibroblast morphology. All cultures underwent further extensive characterization via flow cytometry and immunofluorescence. They were found to be predominantly a myofibroblast-rich population (99% expressing αSMA) as described previously by us and others (15, 27–30). They expressed αSMA, CD90, fibroblast surface protein, and collagen type I. No contaminating cells were found. Immunostaining for macrophages (CD68), T cells (CD3), and progenitor cells (CD34) was negative. There was no evidence of cobblestone-shaped epithelial cells among the cultures. Results of this detailed characterization have been shown previously (27, 30).

### LXA_4_

LXA_4_ [5(S),6(R)-Lipoxin A_4_] (Cayman Chemical) was used at concentrations of 10^−10^ and 10^−8^ mol.

### Flow cytometry

HLMFs were grown in T25 flasks and serum-starved for 24 h prior to experiments. The myofibroblasts were incubated for 24 h and either left unstimulated or stimulated with TGF-β1 (10 ng/ml) (R&D Systems, Oxford, U.K.).

To determine the expression of the LXA_4_ receptor ALXR, HLMFs were detached using 0.1% trypsin/0.1% EDTA and gated using fibroblast surface Ag Thy-1 (Merck, Hertfordshire, U.K.). Myofibroblasts were then labeled with allophycocyanin-conjugated mouse monoclonal anti–formyl-peptide receptor-like 1 (FPRL1; also known as ALXR) (R&D Systems) or allophycocyanin -conjugated isotype control IgG2b Ab (R&D Systems).

To study the inhibitory effects of LXA_4_ on αSMA expression, HLMFs were incubated in the presence of serum-free medium alone or 0.1% ethanol vehicle control or LXA_4_ at 10^−10^ and 10^−8^ mol. Cells were detached using 0.1% trypsin/0.1% EDTA, washed, then fixed, and permeabilized in 4% paraformaldehyde plus 0.1% saponin (Sigma-Aldrich, Poole, Dorset, U.K.) for 20 min on ice. Myofibroblasts were labeled with either: FITC-conjugated mouse monoclonal anti-αSMA (Sigma-Aldrich) or isotype control FITC-conjugated mouse IgG_2a_; secondary Abs labeled with FITC were applied if appropriate. Analysis was performed using single-color flow cytometry on an FACScan (BD Biosciences, Oxford, U.K.).

### Immunofluorescence

HLMFs were grown on eight-well chamber slides and serum-starved for 24 h prior to the experiment. The cells were left unstimulated and incubated in the presence of 0.1% ethanol vehicle control or LXA_4_ at 10^−10^ and 10^−8^ mol for 48 h. Cells were then immunostained as described previously (27) using FITC-conjugated mouse monoclonal anti-αSMA (F3777; 10 μg/ml, Sigma-Aldrich) and isotype control FITC-conjugated mouse IgG_2a_ (X0933, 10 μg/ml; DakoCytomation, Ely, U.K.). Cells were mounted with fluorescent mounting medium and coverslipped. Original images were captured on an epifluorescent microscope (Olympus BX50; Olympus UK, Southend-on-Sea, U.K.); grayscale intensity was examined using Cell F imaging software (Olympus UK). Matched exposures were used for isotype controls.

Actin stress fibers were calculated using a specialized macro on Image J (National Institutes of Health) designed by Dr. Kees Straatman, University of Leicester (15). The macro is capable of providing a quantitative, unbiased score of the number of stress fibers per individual cell by determining the fluctuations of grayscale intensity created by the αSMA staining within the stress fibers.

### Collagen gel contraction assay

HLMFs were serum-starved for 24 h and then pretreated for 24 h with serum-free medium alone, 0.1% ethanol control, LXA_4_ 10^−10^, or LXA_4_ 10^−8^ mol. Cells were detached and embedded in collagen gels as described previously (31). TGF-β1 was then added to appropriate wells to a final concentration of 10 ng/ml. Photographs were taken at 0 and 24 h. The surface area was measured at each time point using ImageJ software (National Institutes of Health; http://rsbweb.nih.gov/ij/).

### Smad2/3 nuclear localization

HLMFs were grown on eight-well chamber slides and serum-starved for 24 h prior to the experiment. The cells were either unstimulated or stimulated with TGF-β1 (10 ng/ml) in the presence of serum-free medium alone, 0.1% ethanol control, LXA_4_ 10^−10^ mol, or LXA_4_ 10^−8^ mol. After 1 h, cells were immunostained using rabbit monoclonal anti-Smad2/3 (0.174 μg/ml; Cell Signaling Technology). Secondary Ab labeled with FITC (F0313; DakoCytomation) was applied and the cells counterstained with DAPI (Sigma-Aldrich). Cells were mounted with fluorescent mounting medium and coverslipped. Images were analyzed as above. The intensity of nuclear Smad2/3 staining was quantified by measuring the grayscale intensity of DAPI-positive nuclei to whole-cell staining.

### Quantitative RT-PCR

HLMF RNA was isolated using the RNeasy Plus Kit (Qiagen, West Sussex, U.K.) according to the manufacturer’s instructions. Primers were designed for αSMA (ACT2A): forward, 5′-TTCAATGTCCCAGCCATGTA-3′ and reverse, 5′-GAAGGAATAGCCACGCTCAG, product size 222 bp from the National Center for Biotechnology Information Reference sequence NM_001141945.1; collagen type I (COL1A1) forward, 5′-TTCTGCAACATGGAGACTGG and reverse, 5′-CGCCATACTCGAACTGGAATC; and collagen type IV (COL4A1), forward, 5′-GGACTACCTGGAACAAAAGGG and reverse, 5′-GCCAAGTATCTCACCTGGATCA, product size 240 bp from reference sequence NM_001845.4; β-actin primers were analyzed using gene-specific Quantitect Primer Assay primers (Qiagen), HS_ACTB_1_SG. All expression data were normalized to β-actin and corrected using the reference dye ROX. Gene expression was quantified by real-time PCR using the Brilliant SYBR Green QRT-PCR 1-Step Master Mix (Stratagene). PCR products were run on a 1.5% agarose gel to confirm the product amplified was the correct size, and each of the products was sequenced to confirm the specificity of the primers.

### Collagen secretion assay

HLMFs were cultured in serum-free medium alone or 0.1% ethanol control and stimulated with TGF-β1 10 ng/ml in the presence of ethanol control, and LXA_4_ at 10^−10^ or 10^−8^ mol for 24 h. Soluble collagen released by HLMFs was quantified using the Sircol collagen assay (Biocolor, County Antrim, U.K.) according to the manufacturer’s instructions (27, 32, 33).

### Proliferation assay

HLMFs were seeded into six-well plates, and when 50%, confluent cells were serum starved for 24 h in serum-free medium. Cells were then stimulated with serum-free medium plus 0.1% ethanol, 10% FBS medium plus 0.1% ethanol, or 10% FBS plus LXA_4_ at 10^−10^ and 10^−8^ mol. After 48 h, cells were mobilized with 0.1% trypsin/0.1% EDTA and counted using a standard hemocytometer. Cell viability was assessed by trypan blue exclusion. Results were counted by two blinded observers with high agreement (intraclass correlation of 0.969). All conditions were performed in duplicate.

### Western blot for Smad proteins

HLMFs were grown in T75 flasks, serum-starved for 24 h, and stimulated with TGF-β1 (10 ng/ml) in the presence of either serum-free medium alone, 0.1% ethanol control, LXA_4_ 10^−10^ mol, or LXA_4_ 10^−8^ mol for 1 h. HLMFs were detached with 0.1% trypsin/EDTA and washed. Protein was isolated using the RIPA buffer lysis system (Santa Cruz Biotechnology), and total protein concentration was determined using the DC Bio-Rad protein Assay (Bio-Rad). A total of 30 μg protein was resolved using 10% Mini-Protean TGX precast gels (Bio-Rad) and then transferred to an Immunobilon-P polyvinylidene difluoride membrane using Transblot Turbo transfer packs (Bio-Rad). Membranes were blocked with 5% milk and incubated with rabbit monoclonal anti-phosphorylated-Smad2/3 (0.231 μg/ml; Cell Signaling Technology) or rabbit monoclonal anti-Smad2/3 (0.0087 μg/ml; Cell Signaling Technology). Protein bands were identified by HRP-conjugated secondary Ab and ECL reagent (Amersham). Immunolabeled proteins were visualized using ImageQuant LAS 4000 (GE Healthcare Life Sciences).

### Statistical analysis

Experiments from an individual donor were performed either in duplicate or triplicate, and a mean value was derived for each condition. Data distribution across donors was tested for normality using the Kolmogorov-Smirnov test. For parametric data, the one-way ANOVA or repeated-measures ANOVA for across-group comparisons was used followed by the appropriate multiple-comparison post hoc test; otherwise an unpaired or paired *t* test was used. For nonparametric data, the Friedman test was used for across group comparisons followed by the appropriate multiple-comparison post hoc test, or the Wilcoxon signed-rank test was used where there were paired groups. GraphPad Prism for windows (version 6; GraphPad Software, San Diego, CA) was used for these analyses. A *p* value < 0.05 was taken to assume statistical significance, and data are represented as mean (± SEM) or median (interquartile ratio).

## Results

### HLMFs express LXA_4_ receptors

LXA_4_ acts via activation of the ALXR G-protein–coupled receptor (also known as FPR2, FPRL1, FPRH1, RFP, and HM63) in low-nanomole concentrations. The functions of this receptor, however, are cell specific; in neutrophils, LXA_4_-ALXR interactions inhibit migration, but in monocytes, these can stimulate chemotaxis (34).

**FIGURE 1.**
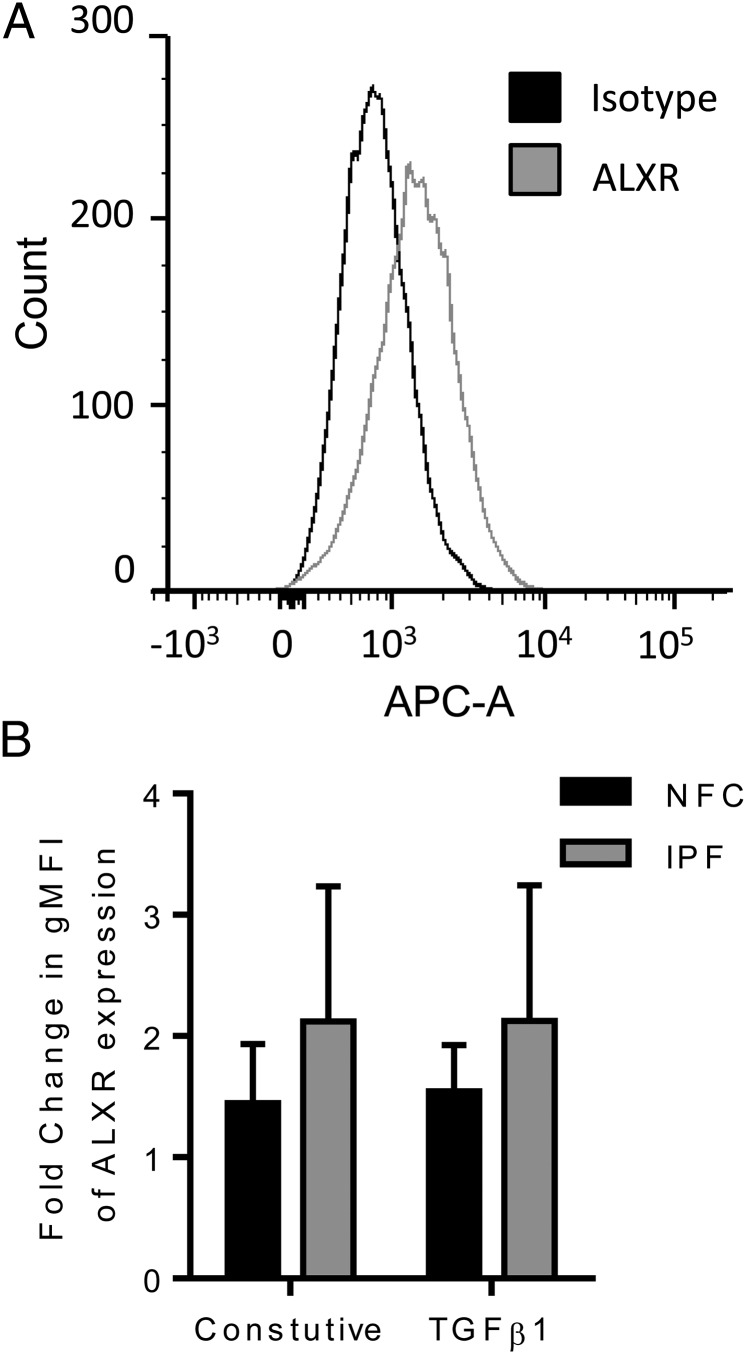
HLMFs express ALXR. (**A**) Representative histogram for ALXR staining assessed by flow cytometry in HLMFs obtained from an IPF donor. (**B**) Both NFC- (*n* = 3) and IPF-derived (*n* = 3) HLMFs expressed ALXR constitutively (*p* = 0.0056, pooled data). ALXR expression was not increased following 24 h of treatment with TGF-β1.

NFC- and IPF-derived HLMFs expressed ALXR (FPRL1) with a whole population shift in comparison with the control Ab (*p* = 0.0056, Mann–Whitney *U* test) (Fig. 1A). There was a trend for a higher expression in the IPF-derived myofibroblasts, although this was not statistically significant, and expression of ALXR did not change following 24 h of TGF-β1 stimulation (Fig. 1B). Thus, HLMFs express LXA_4_ receptors.

### Constitutive αSMA expression and actin stress fiber formation is inhibited by LXA_4_

**FIGURE 2.**
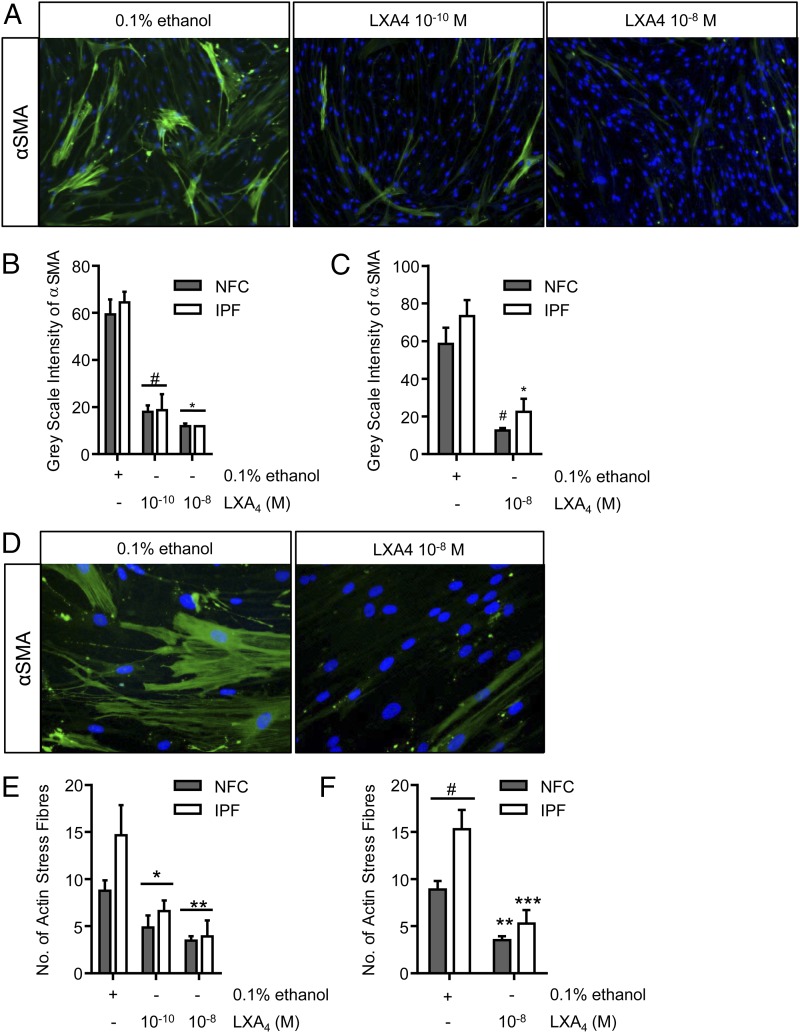
Constitutive αSMA and stress fiber formation is attenuated by LXA_4_. (**A**) Representative immunofluorescent images from an NFC donor showing the decrease in αSMA expression in HLMFs following incubation with LXA_4_ (10^−10^ and 10^−8^ mol). Original magnification ×100. (**B**) αSMA expression was assessed by measuring grayscale intensity. LXA_4_ decreased αSMA expression at 10^−10^ and 10^−8^ mol in NFC-derived and IPF-derived HLMFs (*n* = 6 and *n* = 3, respectively, data pooled) (*p* < 0.0001, repeated-measures ANOVA, ^#^*p* = 0.0001, *p < 0.0001, corrected by Sidak multiple-comparison test). For each donor, a minimum of 10 cells per field were measured and the mean taken. (**C**) In both NFC- (*n* = 6) and IPF-derived (*n* = 6) HLMFs, αSMA was attenuated by LXA_4_ 10^−8^ mol (two-way ANOVA corrected by Sidak multiple-comparison test; NFC, ^#^*p* = 0.0004; IPF, *p = 0.0002). (**D**) Cells were noticeably more spindle-like following treatment with LXA_4_; a representative image from an IPF donor is shown. Original magnification ×200. (**E**) An ImageJ macro (National Institutes of Health) was used to quantify the number of actin filaments, and a minimum of 10 cells from each donor was assessed and averaged. LXA_4_ decreased the number of actin filaments at both 10^−10^ and 10^−8^ mol in both NFC- and IPF-derived cells (*n* = 6 and *n* = 4, respectively, data pooled) (*p* = 0.0003, repeated-measures ANOVA, *p = 0.0135, **p = 0.0005, corrected by Sidak multiple-comparison test). (**F**) IPF-derived HLMFs expressed increased numbers of SMA filaments (stress fibers) in comparison with NFC-derived cells (^#^*p* = 0.0177). Actin filaments in both NFC- (*n* = 6) and IPF-derived (*n* = 6) HLMFs were attenuated by LXA_4_ 10^−8^ mol (two-way ANOVA corrected by Sidak multiple-comparison test; NFC, **p = 0.0005; IPF, ***p < 0.0001).

We and others have shown that fibroblasts obtained from human lung parenchyma express high quantities of αSMA and have the typical myofibroblast contractile phenotype (27, 29, 35, 36). Furthermore, these HLMFs derived from IPF donors express higher numbers of actin stress fibers constitutively than cells from NFC donors (15). After 48 h of treatment with LXA_4_ (10^−10^ and 10^−8^ mol), the amount of constitutive αSMA staining was dose dependently reduced (Fig. 2A–C). Following treatment with LXA_4_ cells were less stellate and became more spindle like, a similar morphology to inactivated fibroblasts (Fig. 2D). IPF-derived HLMFs again expressed more αSMA stress fibers at baseline in comparison with NFC-derived cells (*p* = 0.0177). LXA_4_ reduced the number of actin stress fibers in both IPF- and NFC-derived cells and to a similar extent (Fig. 2E, 2F).

### Constitutive HLMF contraction is inhibited by LXA_4_

**FIGURE 3.**
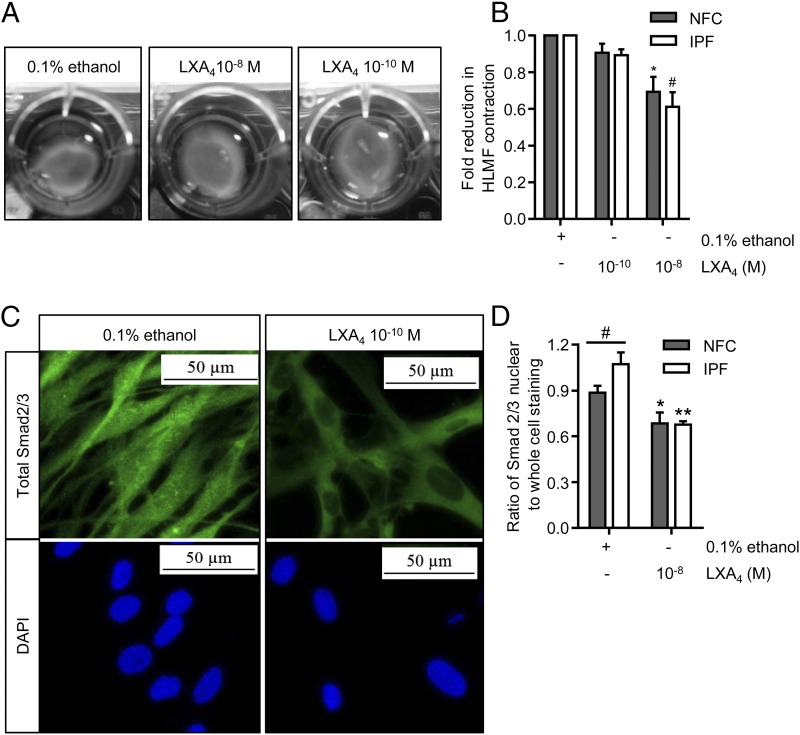
Basal HLMF contraction and Smad2/3 nuclear translocation are inhibited by LXA_4_. (**A**) Representative images from an NFC donor showing the spontaneous contraction of HLMFs within a collagen gel and its suppression by LXA_4_. (**B**) Constitutive HLMF contraction was significantly attenuated by LXA_4_ (10^−8^ mol) in both NFC- (*n* = 6) and IPF-derived (*n* = 5) cells (two-way ANOVA corrected by Sidak multiple-comparison test; NFC, *p = 0.0009; IPF, ^#^*p* = 0.0001). (**C**) Representative images from an IPF donor displaying total Smad2/3 expression within HLMFs. At baseline, the HLMFs have staining both in the nucleus and within the cytoplasm, which is reduced upon incubation with LXA_4_ (10^−8^ mol) for 1 h. (**D**) The ratio of total Smad2/3 nuclear to whole-cell staining was assessed by measuring grayscale intensity. IPF-derived (*n* = 6) HLMFs demonstrated increased expression of total Smad2/3 within the nucleus in comparison with NFC donors (*n* = 6) (^#^*p* = 0.043, Mann–Whitney *U* test). LXA_4_ (10^−8^ mol) significantly attenuated the amount of Smad2/3 localized to the nucleus in both NFC- and IPF-derived cells (two-way ANOVA corrected by Sidak multiple-comparison test; NFC, *p = 0.0254; IPF, **p = 0.0006).

One of the main features of a myofibroblast is its ability to drive tissue repair and wound closure by reorganizing the ECM via contraction (37). We therefore investigated the effects of LXA_4_ on constitutive HLMF contraction. HLMF contraction was examined over 24 h in the presence of LXA_4_ at 10^−10^ and 10^−8^ mol using collagen gels (Fig. 3A). With both NFC- and IPF-derived HLMFs, basal contraction was dose dependently reduced by LXA_4_ (*p* = 0.0009 and *p* = 0.0007, respectively, for LXA_4_ at 10^−8^ mol) (Fig. 3B).

### Constitutive HLMF Smad2/3 nuclear translocation is inhibited by LXA_4_

It has previously been reported that αSMA expression and stress fiber formation in myofibroblasts is regulated in part by the TGF-β1/Smad signaling pathway (10, 11). As LXA_4_ inhibited both constitutive αSMA expression and HLMF contraction, we examined whether LXA_4_ disrupts constitutive Smad2/3 nuclear translocation using immunofluorescent staining. HLMFs had detectable Smad2/3 within the nucleus at baseline, and the IPF-derived HLMFs displayed a higher proportion of nuclear staining in comparison with the NFC-derived cells (*p* = 0.043), in keeping with previous work (15). This nuclear Smad2/3 immunostaining was reduced and to a similar extent in both IPF- and NFC-derived HLMFs in the presence of LXA_4_ (10^−8^ mol) for 1 h (Fig. 3C, 3D).

### TGF-β1–induced αSMA expression and contraction are inhibited by LXA_4_ in HLMFs

**FIGURE 4.**
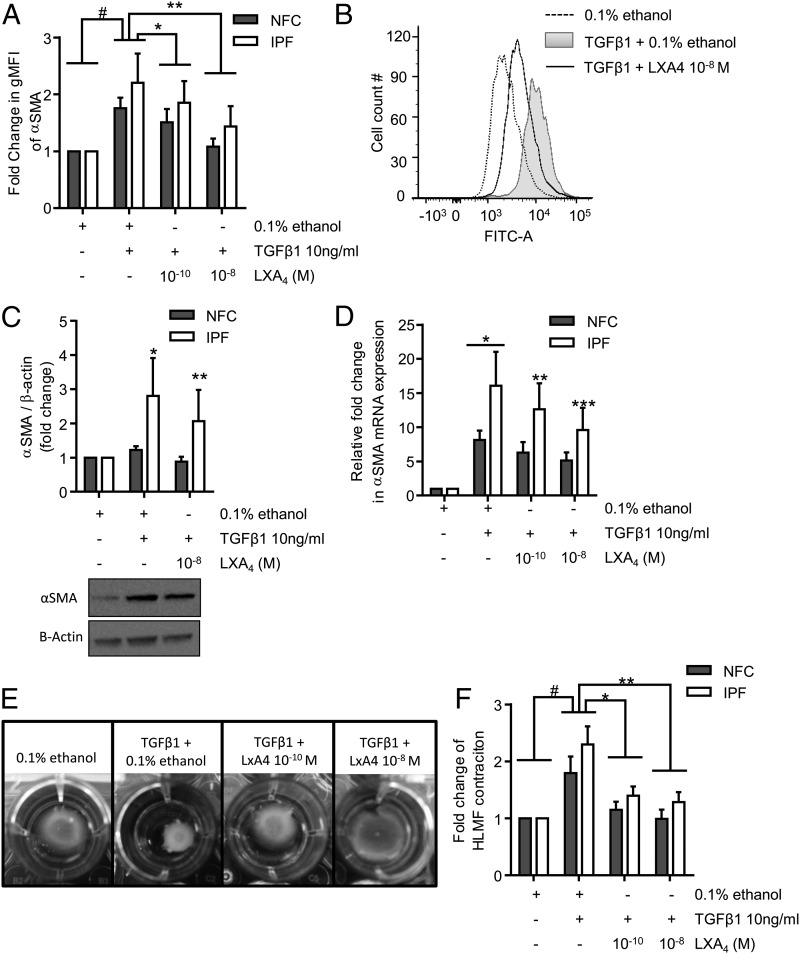
TGF-β1–dependent increases in αSMA mRNA and protein expression and HLMF contraction are attenuated by LXA_4_. (**A**) Flow cytometry was used to assess the changes in αSMA expression following TGF-β1 (10 ng/ml) stimulation in the presence of LXA_4,_ in both NFC- (*n* = 4) and IPF-derived (*n* = 3) HLMFs. αSMA expression in HLMFs was significantly increased following TGF-β1 stimulation (^#^*p* = 0.0070, paired *t* test). LXA_4_ significantly attenuated TGF-β1–dependent increases in αSMA expression at 10^−10^ and 10^−8^ mol (*p = 0.011 and **p = 0.008, respectively; repeated-measures ANOVA corrected by Sidak multiple-comparison test, statistics were performed on pooled data). (**B**) Representative fluorescent histogram showing αSMA expression in HLMFs under the above conditions. (**C**) Densitometry results of Western blot analysis indicating the increased αSMA expression following TGF-β1 (10 ng/ml) stimulation for 24 h in IPF donors (*p = 0.0344, paired *t* test). This increase was significantly attenuated by LXA4 10^−8^ mol (**p = 0.0274, paired *t* test). No significant difference was found between NFC- and IPF-derived HLMFs in terms of TGF-β1–stimulated αSMA expression (*p* = 0.25). (**D**) Quantitative PCR results displaying the relative expression of αSMA in HLMFs following 24 h of stimulation with TGF-β1 (10 ng/ml) in the presence of 0.1% ethanol or LXA_4_ at 10^−10^ and 10^−8^ mol. TGF-β1 significantly upregulated αSMA mRNA expression in both NFC- (*n* = 6) and IPF-derived (*n* = 5) HLMFs (*p* = 0.018, one-sample *t* test). IPF-derived HLMFs demonstrated a significantly greater increase in mRNA expression in comparison with NFC-derived cells (*p = 0.0261). TGF-β1–dependent upregulation of αSMA mRNA expression was significantly inhibited in IPF-derived cells in the presence of LXA_4_ 10^−10^ and 10^−8^ mol (**p = 0.0479, ***p = 0.0033, respectively, two-way ANOVA, corrected by Sidak multiple-comparison test). (**E**) Representative images from an IPF donor showing the TGF-β1–stimulated contraction of HLMFs within a collagen gel and its suppression by LXA_4_. (**F**) HLMFs from both NFC- (*n* = 6) and IPF-derived (*n* = 5) donors displayed increased contraction following TGF-β1 (10 ng/ml) stimulation (^#^*p* = 0.0026, one-sample *t* test, pooled data). LXA_4_ significantly attenuated HLMF contraction in both NFC- (*n* = 6) and IPF-derived (*n* = 5) myofibroblasts at 10^−10^ and 10^−8^ mol (*p* = 0.0005, repeated-measures ANOVA, *p = 0.0057, **p = 0.0006, corrected by Dunn multiple-comparison test).

Next, we investigated whether LXA_4_ inhibits TGF-β1–induced profibrotic activity in HLMFs. Cells were treated with TGF-β1 in the presence of 0.1% ethanol control or LXA_4_ at 10^−10^ and 10^−8^ mol, and flow cytometry was performed. In comparison with vehicle control, TGF-β1 stimulation significantly increased αSMA protein expression in both NFC- and IPF-derived HLMFs (*p* = 0.0070) (Fig. 4A). The TGF-β1–dependent increase in αSMA expression was dose dependently inhibited in both NFC- and IPF-derived HLMFs by LXA_4_ at 10^−10^ mol (*p* = 0.0106) and 10^−8^ mol (*p* = 0.0076) (Fig. 4A, 4B). This was also confirmed by Western blot analysis, in which TGF-β1 increased αSMA expression in IPF-derived HLMFs but not NFC-derived cells, and which was significantly attenuated by LXA_4_ at 10^−8^ mol (*p* = 0.0274) (Fig. 4C).

Similarly, TGF-β1–dependent stimulation increased αSMA mRNA expression in HLMFs, which was significantly greater in IPF-derived HLMFs compared with NFC-derived cells (*p* = 0.0261). This TGF-β1–dependent αSMA mRNA expression was significantly reduced in IPF-derived HLMFs following treatment with LXA_4_ at 10^−10^ and 10^−8^ mol (*p* = 0.0479 and *p* = 0.0033, respectively, two-way ANOVA) (Fig. 4D), but not NFC-derived cells.

To study the effects of LXA_4_ on TGF-β1–dependent HLMF contraction, HLMFs were cultured within collagen gels and their contraction monitored over 24 h. Pictures were taken at 0 and 24 h and the contraction measured using ImageJ software (National Institutes of Health); representative images are displayed in Fig. 4E. TGF-β1 stimulation increased HLMF contraction in comparison with vehicle control (0.1% ethanol) (*p* = 0.0026), with no differences in response between NFC- and IPF-derived cells (Fig. 4F). LXA_4_ significantly reduced TGF-β1–dependent HLMF contraction at both 10^−10^ mol (*p* = 0.0057) and 10^−8^ mol (*p* = 0.0006). Thus, LXA_4_ not only inhibits constitutive HLMF αSMA expression and contraction, but also the increased αSMA expression and contraction induced by the potent profibrotic mediator TGF-β1 (38, 39).

### TGF-β1–induced collagen mRNA expression and collagen secretion is inhibited by LXA_4_

**FIGURE 5.**
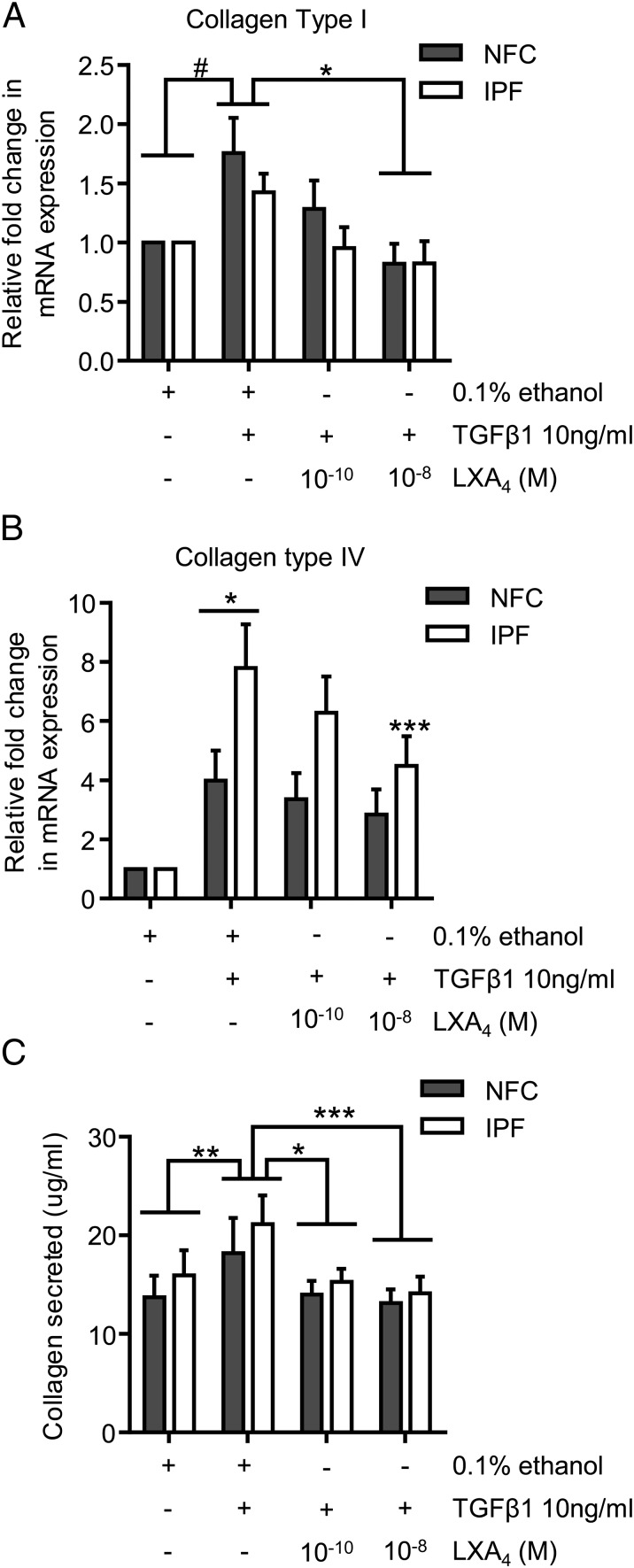
LXA4 significantly decreases TGF-β1–dependent increases in collagen mRNA expression and collagen secretion. (**A**) Collagen type I mRNA expression was significantly upregulated following 24 h of TGF-β1 stimulation in NFC- and IPF-derived HLMFs (*n* = 6 and *n* = 5, respectively) (^#^*p* = 0.0064, one-sample *t* test on pooled data). LXA_4_ significantly reduced TGF-β1–dependent upregulation of collagen type I mRNA at 10^−8^ mol (*p* = 0.0214, one-way ANOVA, *p = 0.0057, corrected by Sidak multiple-comparison test). (**B**) Collagen type IV mRNA expression was significantly upregulated following 24 h of TGF-β1 stimulation in NFC- and IPF-derived HLMFs (*n* = 7 and *n* = 5, respectively) (*p = 0.0031 and ***p < 0.0001, respectively, two-way ANOVA corrected by Sidak multiple-comparison test). The TGF-β1–dependent increase in collagen type IV mRNA expression was significantly greater in IPF-derived HLMFs in comparison with NFC-derived cells (*p = 0.0480, unpaired *t* test). The TGF-β1–dependent increase in collagen type IV mRNA expression in IPF HLMFs was significantly reduced by LXA_4_ at 10^−8^ mol, **p = 0.0039, two-way ANOVA, corrected by Sidak multiple-comparison test). (**C**) The amount of collagen secreted by both NFC- and IPF-derived HLMFs increased significantly following TGF-β1 (10 ng/ml) for 24 h, with no difference between NFC and IPF (**p = 0.0022 on pooled data; NFC, *n* = 4 and IPF, *n* = 4). LXA_4_ significantly inhibited collagen secretion at both 10^−10^ and 10^−8^ mol (*p = 0.0015, ***p = 0.0002, two-way ANOVA, pooled data).

To further elucidate the inhibitory effects of LXA_4_ we investigated mRNA expression of collagen type I and type IV, which are two of the most abundant collagens found within the IPF lungs (39, 40). Collagen type I mRNA expression was significantly increased by TGF-β1 in both NFC- and IPF-derived HLMFs compared with control (*p* = 0.0064, one-sample *t* test, pooled data) (Fig. 5A). This increase was significantly reduced by LXA_4_ at 10^−8^ mol in both NFC- and IPF-derived cells (*p* = 0.0057, one-way ANOVA corrected by Dunn multiple-comparison test) (Fig. 5A). Similarly, collagen type IV mRNA expression was significantly increased in both NFC- and IPF-derived HLMFs (*p* = 0.0031 and *p* < 0.0001, respectively, two-way ANOVA corrected by Dunnett multiple-comparison test). IPF-derived HLMFs expressed significantly more collagen type IV mRNA following TGF-β1 stimulation in comparison with NFC-derived cells (*p* = 0.0480, unpaired *t* test) (Fig. 5B). LXA_4_ dose dependently inhibited TGF-β1–induced collagen type IV mRNA expression in IPF-derived cells at 10^−8^ mol (*p* = 0.0039, two-way ANOVA corrected by Sidak multiple-comparison test).

The ability of LXA_4_ to downregulate TGF-β1–increased collagen mRNA was paralleled by a reduction in total collagen secretion measured using the Sircol assay. TGF-β1 significantly increased the amount of total collagen secreted by NFC- and IPF-derived cells (*p* = 0.0022), which was dose-dependently inhibited by LXA_4_ (10^−10^ mol, *p* = 0.0015; 10^−8^ mol, *p* = 0.0002) (Fig. 5C).

### FBS-induced HLMF proliferation is attenuated by LXA_4_

**FIGURE 6.**
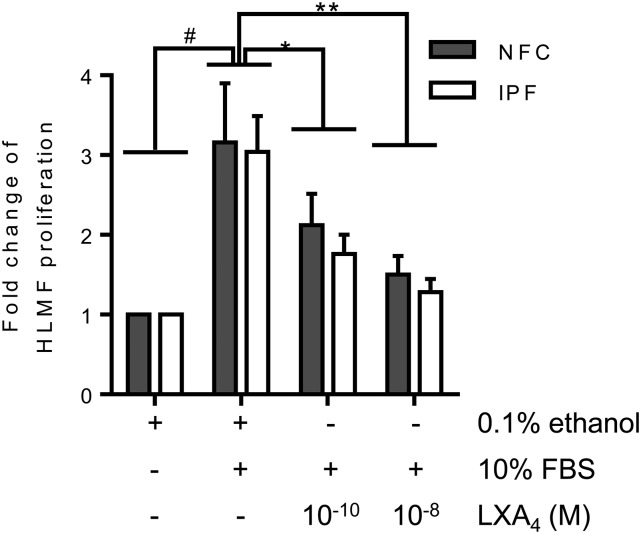
FBS-dependent HLMF proliferation is inhibited by LXA_4_. HLMF proliferation was increased following 48 h of stimulation with FBS and to a similar extent in both NFC- and IPF-derived HLMFs (^#^*p* = 0.0006, one-sample *t* test, pooled data; NFC, *n* = 5, and IPF, *n* = 5). This increase in HLMF proliferation was significantly reduced with LXA_4_ at both 10^−10^ and 10^−8^ mol (*p* = 0.0001, repeated-measures ANOVA, *p = 0.0001, **p = 0.001, respectively, corrected by Dunn multiple-comparison test).

HLMF proliferation was assessed after 48 h of stimulation with 10% FBS, which significantly increased proliferation (*p* = 0.0006) (Fig. 6). In both NFC- and IPF-derived HLMFs, FBS-dependent proliferation was significantly decreased by LXA_4_ at 10^−10^ mol (*p* = 0.0001) and 10^−8^ mol (*p* = 0.001) (Fig. 6). In summary, FBS-induced HLMF proliferation is attenuated by treatment with LXA_4._

### TGF-β1–dependent Smad2/3 nuclear translocation is disrupted by LXA_4_

**FIGURE 7.**
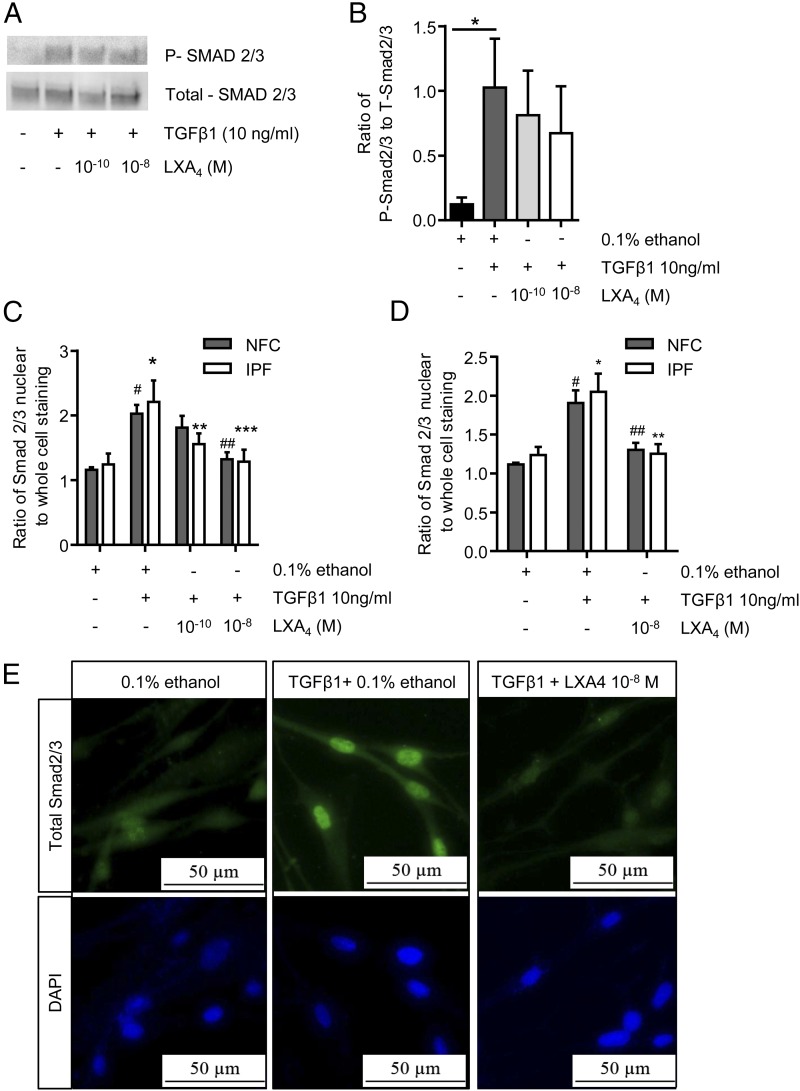
LXA_4_ does alter Smad2/3 phosphorylation but exerts its effect through inhibition of Smad2/3 translocation to the nucleus. (**A** and **B**) Using Western blot analysis, we investigated the effect of LXA_4_ on TGF-β1–induced phosphorylation of Smad2/3; a representative image from an NFC donor is shown. TGF-β1 significantly increased Smad2/3 phosphorylation in HLMFs (*p = 0.0110; NFC, *n* = 2; and IPF, *n* = 3). However, LXA_4_ at 10^−10^ and 10^−8^ mol had no significant effect on the TGF-β1–dependent increase in Smad2/3 phosphorylation in either NFC- or IPF-derived HLMFs. (**C**) Immunofluorescent analysis of total Smad2/3 nuclear translocation showed a significant increase in both NFC (*n* = 5) and IPF (*n* = 4) HLMFs (^#^*p* = 0.0007, *p = 0.0007, respectively). This TGF-β1–dependent increase in total nuclear translocation was significantly and dose dependently attenuated by LXA_4_ (two-way ANOVA corrected by Sidak multiple-comparison test; NFC, 10^−8^ mol, ^##^*p* = 0.0051; IPF, 10^−10^ mol, **p = 0.0208 and 10^−8^ mol, ***p = 0.0011). (**D**) In both NFC- (*n* = 6) and IPF-derived (*n* = 6) HLMFs, TGF-β1–dependent total Smad2/3 nuclear translocation was attenuated by LXA_4_ 10^−8^ mol (two-way ANOVA corrected by Sidak multiple-comparison test; NFC, ^#^*p* = 0.0003, ^##^*p* = 0.00037; IPF, *p = 0.0002, **p = 0.0003). (**E**) Representative immunofluorescent images from an NFC donor demonstrating the increased nuclear translocation of total Smad2/3 following TGF-β1 stimulation and the inhibition of this by LXA_4_ (10^−8^ mol).

The ability of LXA_4_ to attenuate both constitutive Smad2/3 nuclear localization and several TGF-β1–dependent cell processes in HLMFs suggested that LXA_4_ may also attenuate TGF-β1–dependent Smad2/3 nuclear translocation. Phosphorylation is a key initial event in the activation of these Smad proteins. Using Western blot analysis, we therefore investigated the effect of LXA_4_ on TGF-β1–induced phosphorylation of Smad2/3 and the expression of total Smad2/3 in HLMFs. As previously reported in many studies, TGF-β1 significantly increased Smad2/3 phosphorylation in HLMFs (*p* = 0.0110). However, LXA_4_ at 10^−10^ and 10^−8^ mol had no significant effect on the TGF-β1–dependent increase in Smad2/3 phosphorylation in either NFC- or IPF-derived HLMFs (Fig. 7A, 7B).

Once TGF-β1 has induced phosphorylation of Smad2/3, they interact with Smad4, and this complex translocates to the nucleus to initiate gene transcription. Although Smad2/3 phosphorylation was not inhibited, TGF-β1–dependent Smad2/3 nuclear translocation was significantly attenuated by LXA_4_ (Fig. 7C–E). Thus LXA_4_ appears to significantly disrupt TGF-β1–dependent nuclear translocation of Smad2/3, but not Smad2/3 phosphorylation.

## Discussion

The myofibroblast is implicated as the key cell driving the progression of IPF through the synthesis of excess fibrotic extracellular matrix and tissue contraction. The myofibroblast is therefore an attractive target for novel antifibrotic therapies. In this study, we have demonstrated that HLMFs express LXA_4_ receptors and that LXA_4_ attenuates: 1) constitutive HLMF αSMA expression, stress fiber formation, contraction, and Smad2/3 nuclear localization; 2) TGF-β1–dependent increases in HLMF αSMA expression, contraction, collagen mRNA expression, collagen secretion, and Smad2/3 nuclear localization; and 3) serum-dependent HLMF proliferation.

Fibroblasts and myofibroblasts demonstrate marked functional and phenotypic heterogeneity between tissues and across species. As an example, there is a marked difference in the phenotype of fibroblasts grown from human airway and lung parenchyma, with those from lung spontaneously displaying a myofibroblast phenotype (29, 35, 36). When considering novel therapeutic targets, it is therefore important to study cells from the species, tissue compartment and disease of interest. Expression of the LXA_4_ receptor ALXR (FPRL1) has been demonstrated previously in human synovial fibroblasts by RT-PCR (41), and these cells and rat fibroblast cell lines respond to LXA_4_ implying functional receptor expression (22–41). In this study, we have demonstrated that ALXR protein is expressed on the surface of the majority of parenchymal HLMFs derived from both NFC and IPF tissue.

In a rat fibroblast cell line, LXA_4_ at 10^−9^ mol attenuated TGF-β1–dependent Smad2 phosphorylation (but not Smad3 phosphorylation) and MAPK activation, and inhibited TGF-β1–dependent gene transcription (22). We also found that LXA_4_ inhibited TGF-β1–dependent gene transcription, but in contrast to Börgeson et al. (22), we could not detect inhibition of Smad2/3 phosphorylation, although there was reduced TGF-β1–dependent nuclear Smad2/3 translocation. The means by which LXA_4_ would prevent nuclear translocation of activated Smads without affecting phosphorylation is intriguing and will require further work to elucidate the mechanism. Nevertheless, the ability of LXA_4_ to inhibit Smad nuclear localization is in keeping with its ability to inhibit TGF-β1–dependent gene transcription and the downstream production of collagen and αSMA in HLMFs.

Several phenotypic differences have been observed previously between control and IPF-derived HLMFs in culture, suggesting that there may be genetic and/or epigenetic changes that promote a profibrotic HLMF phenotype in patients who develop IPF. Interestingly, we found that LXA_4_ not only reduced TGF-β1–dependent profibrotic responses, but promoted HLMF differentiation toward a fibroblast phenotype by reducing constitutive αSMA actin expression and actin stress fiber formation. This in turn is likely to explain the reduced constitutive HLMF contraction evident in collagen gels. This occurred in conjunction with a reduction in nuclear Smad2/3 immunostaining, which occurred within 1 h of LXA_4_ exposure, suggesting there is a component of constitutive Smad2/3 signaling in HLMFs at rest. This ability of LXA_4_ to promote dedifferentiation toward a fibroblast phenotype suggests that if there is a degree of constitutive profibrotic HLMF activity in IPF lungs due to genetic/epigenetic factors, LXA_4_ or a stable analog might be a particularly useful therapy.

In addition to the inhibition of TGF-β1–dependent stimulation, we found that LXA_4_ also inhibited HLMF proliferation induced by serum, and this is in keeping with previous work showing that LXA_4,_ inhibited connective tissue growth factor–dependent proliferation of a human fibroblast cell line (25). Previous studies using LXA_4_ in cell-culture systems suggest it is highly active in the nanomole range. Our data are consistent with this, with effects readily evident in HLMFs at 10^−10^ mol (0.1 nmol), although more consistent at 10^−8^ mol.

In summary, we have shown that LXA_4_ inhibits many TGF-β1–dependent profibrotic responses in healthy and IPF-derived HLMFs, which may result from the inhibition of Smad2/3 nuclear translocation. Furthermore, LXA_4_ promotes HLMF dedifferentiation in the resting state, suggesting it may have the potential to reverse the fibrotic process. In support of this, and of particular relevance to this study, a stable epi-LXA4 analog markedly inhibited bleomycin-induced pulmonary fibrosis in mice when administered either preventively or curatively, with reversal of fibrosis evident in the latter (26). This was associated with a reduction in the accumulation and differentiation of myofibroblasts in the lung parenchyma. Thus, there are consistent in vitro and in vivo data from primary HLMFs and the mouse bleomycin model, respectively, indicating that LXA_4_ or its stable analogs may not only prevent the progression of lung fibrosis, but also potentially reverse it. This indicates that clinical trials of LXA_4_ analogs should be considered in patients with IPF.
